# Supplementary material for: Mineral Nutrition of Naturally Growing Scots Pine and Norway Spruce under Limited Water Supply
Source: Plants (Basel). 2022 Oct 9;11(19):2652. doi: 10.3390/plants11192652 (PMC9573269; doi:10.3390/plants11192652)
Supplement: Supplementary file 1 [file plants-11-02652-s001.zip › Table S1.pdf]

Table S1. pH and contents of nutrients at 4 depths (I – 5-15 cm, II – 25-35 cm, III – 60-70 cm, and IV – 110-120 cm) for pine-inhabited Site III (arid) and Site II (normal) and for spruce-inhabited Site II (arid) and Site (I) (normal).

Parameter	Depth, cm	Pine stands				Spruce stands			
		Normal (Site II)		Arid (Site III)		Normal (Site I)		Arid (Site II)	
		N3	N24	A3	A24	N3	N24	A3	A24
pH	5-15	4.24±0.11	3.88±0.06	4.44±0.08	4.24±0.04	3.46±0.03	3.74±0.27	4.24±0.11	3.88±0.06
	25-35	4.70±0.07	4.58±0.02	4.88±0.05	4.57±0.05	3.72±0.03	3.69±0.02	4.70±0.07	4.58±0.02
	60-70	4.77±0.05	4.66±0.03	4.80±0.05	4.48±0.05	4.17±0.07	4.12±0.03	4.77±0.05	4.66±0.03
	110-120	ND	4.28±0.07	ND	4.61±0.06	ND	4.27±0.08	ND	4.28±0.07
K, mg/kg	5-15	1.53±0.20	6.02±0.39	3.89±0.59	6.45±0.74	6.29±0.95	5.79±0.24	1.53±0.20	6.02±0.39
	25-35	1.22±0.28	2.71±0.24	2.30±0.43	4.93±0.98	4.38±0.36	4.69±0.44	1.22±0.28	2.71±0.24
	60-70	1.33±0.25	3.05±0.33	2.05±0.49	5.59±0.82	6.96±1.04	8.05±0.56	1.33±0.25	3.05±0.33
	110-120	ND	3.55±0.47	ND	3.71±0.48	ND	8.88±0.66	ND	3.55±0.47
Ca, mg/kg	5-15	28.6±6.5	38.2±6.9	139.3±19.2	71.4±10.8	11.3±3.0	28.1±3.8	28.6±6.5	38.2±6.9
	25-35	34.6±4.5	42.4±4.4	53.5±5.0	39.6±8.1	2.22±0.75	10.5±1.9	34.6±4.5	42.4±4.4
	60-70	27.1±6.7	23.6±1.6	23.0±2.7	32.3±9.4	36.7±9.8	43.2±6.9	27.1±6.7	23.6±1.6
	110-120	ND	14.9±2.8	ND	6.45±2.80	ND	60.5±11.7	ND	14.9±2.8
Mg, mg/kg	5-15	0.57±0.10	2.01±0.34	3.40±0.28	1.68±0.24	2.67±0.43	1.82±0.37	0.57±0.10	2.01±0.34
	25-35	0.52±0.08	0.48±0.06	0.87±0.30	0.88±0.40	0.80±0.18	0.84±0.13	0.52±0.08	0.48±0.06
	60-70	0.43±0.11	0.23±0.06	0.41±0.07	0.68±0.19	2.36±1.02	1.56±0.28	0.43±0.11	0.23±0.06
	110-120	ND	0.27±0.15	ND	0.10±0.03	ND	2.67±1.05	ND	0.27±0.15
P, mg/kg	5-15	1.05±0.40	0.97±0.17	0.54±0.08	0.62±0.07	0.77±0.12	0.81±0.06	1.05±0.40	0.97±0.17
	25-35	6.16±1.31	3.75±0.96	1.13±0.17	0.82±0.10	0.38±0.04	1.30±0.41	6.16±1.31	3.75±0.96
	60-70	5.70±1.59	3.47±0.92	0.61±0.09	1.15±0.11	6.19±0.93	5.76±0.45	5.70±1.59	3.47±0.92
	110-120	ND	2.45±0.45	ND	0.67±0.25	ND	5.88±0.44	ND	2.45±0.45
Fe, mg/kg	5-15	4.87±1.75	22.95±2.30	12.23±3.38	12.40±1.93	4.41±0.83	10.11±3.19	4.87±1.75	22.95±2.30
	25-35	12.96±3.16	6.03±1.17	6.42±0.53	9.77±0.92	3.13±0.43	14.46±3.52	12.96±3.16	6.03±1.17
	60-70	12.45±2.56	15.80±5.58	3.57±0.56	9.27±1.64	21.78±3.65	24.52±1.74	12.45±2.56	15.80±5.58
	110-120	ND	2.80±0.56	ND	1.92±0.49	ND	21.95±2.88	ND	2.80±0.56
Mn, mg/kg	5-15	0.60±0.11	1.68±0.20	3.70±1.18	3.10±0.48	1.23±0.23	0.99±0.20	0.60±0.11	1.68±0.20
	25-35	0.19±0.01	0.10±0.01	0.53±0.08	0.70±0.13	0.41±0.06	2.90±0.90	0.19±0.01	0.10±0.01
	60-70	0.39±0.10	0.08±0.01	0.41±0.04	0.31±0.07	4.31±0.95	7.95±1.10	0.39±0.10	0.08±0.01
	110-120	ND	0.10±0.01	ND	0.15±0.01	ND	3.88±0.75	ND	0.10±0.01

Zn, mg/kg	5-15	0.11±0.01	0.21±0.02	0.15±0.02	0.15±0.04	0.33±0.08	0.20±0.02	0.11±0.01	0.21±0.02
	25-35	0.11±0.03	0.08±0.02	0.03±0.01	0.08±0.02	0.12±0.02	0.14±0.02	0.11±0.03	0.08±0.02
	60-70	0.04±0.01	0.04±0.01	0.02±0.00	0.07±0.03	0.23±0.03	0.23±0.03	0.04±0.01	0.04±0.01
	110-120	ND	0.06±0.01	ND	0.04±0.02	ND	0.51±0.13	ND	0.06±0.01
