# Supplementary material for: Mineral Nutrition of Naturally Growing Scots Pine and Norway Spruce under Limited Water Supply
Source: Plants (Basel). 2022 Oct 9;11(19):2652. doi: 10.3390/plants11192652 (PMC9573269; doi:10.3390/plants11192652)
Supplement: Supplementary file 1 [file plants-11-02652-s001.zip › Table S2.pdf]

Table S2. Nutrient contents in wood, needles and bark of pine and spruce.

Nutrients	Pine Stands				Spruce Stands			
	Normal (Site II)		Arid (Site III)		Normal (Site I)		Arid (Site II)	
	N3	N24	A3	A24	N3	N24	A3	A24
	Pine wood				Spruce wood			
K, mg/g	2.07±0.25	3.31±.25	2.61±0.41	3.15±0.66	1.07±0.17	1.57±0.18	1.90±0.30	2.20±0.23
Ca, mg/g	0.57±0.05	1.48±0.15	0.59±0.03	1.77±0.41	0.82±0.16	1.40±0.13	1.63±.27	2.23±0.29
Mg, mg/g	0.21±0.02	0.50±0.05	0.27±0.02	0.62±0.05	0.15±0.02	0.27±0.03	0.17±0.03	0.41±0.04
P, mg/g	0.69±0.08	0.97±0.07	0.78±0.12	1.04±0.15	0.33±0.05	0.40±0.05	0.51±0.07	0.61±0.06
Fe, μg/g	11.4±2.5	24.0±2.0	3.69±0.88	34.6±3.3	4.46±1.22	7.59±2.64	18.0±5.0	28.2±3.7
Mn, μg/g	77.2±5.5	63.9±6.1	77.4±7.5	40.9±6.7	246.4±30.1	268.5±30.1	142.5±18.0	201.8±24.6
Zn, μg/g	16.9±1.5	33.8±2.9	18.6±2.2	35.4±3.1	22.4±4.0	32.4±3.5	28.6±5.7	53.0±9.4
Cu, μg/g	4.54±0.34	6.00±0.50	4.36±0.42	5.66±0.38	3.45±0.25	4.26±0.26	3.38±0.34	6.38±0.78
	Pine needles				Spruce needles			
K, mg/g	5.11±0.24	5.76±0.09	5.43±0.26	5.94±0.16	5.80±0.31	6.00±0.35	5.27±0.25	5.48±0.19
Ca, mg/g	2.81±0.37	2.25±0.09	1.74±0.13	2.46±0.27	6.39±0.41	5.40±0.30	6.95±0.48	7.04±0.28
Mg, mg/g	0.43±0.01	0.82±0.02	0.52±0.02	0.79±0.02	0.50±0.01	0.89±0.03	0.54±0.01	1.02±0.03
P, mg/g	1.38±0.04	1.35±0.03	1.29±0.02	1.24±0.02	1.36±0.03	1.41±0.04	1.54±0.06	1.28±0.04
Fe, μg/g	78.6±11.3	34.2±0.6	31.9±1.3	44.2±1.9	65.1±4.8	35.7±1.0	45.7±3.0	30.3±1.0
Mn, μg/g	385.7±29.1	187.1±10.3	302.9±25.4	109.6±8.6	1740±174.1	1152±95.1	726.1±64.6	537.5±29.0
Zn, μg/g	40.5±1.5	36.6±1.2	38.4±2.2	37.3±2.0	34.4±1.5	36.2±1.6	39.9±1.3	41.5±2.0
Cu, μg/g	4.12±0.22	4.70±0.20	3.63±0.16	3.74±0.20	2.10±0.06	4.58±0.17	2.63±0.17	3.04±0.09
	Pine bark				Spruce bark			
K, mg/g	4.77±0.53	6.82±0.84	4.28±0.47	5.21±0.50	3.79±0.51	4.68±0.42	5.36±0.58	5.33±0.59
Ca, mg/g	4.45±0.76	4.51±0.44	3.00±0.34	3.75±0.56	7.15±0.53	8.69±0.73	7.43±0.77	7.74±1.00
Mg, mg/g	0.48±0.02	0.77±0.05	0.50±0.04	0.91±0.10	0.52±0.04	1.02±0.06	0.50±0.04	0.85±0.08
P, mg/g	1.21±0.06	1.18±0.08	1.15±0.05	1.18±0.08	1.35±0.09	1.58±0.07	1.32±0.12	1.25±0.07
Fe, μg/g	258.0±59.0	113.8±10.2	76.5±11.7	134.9±31.4	191.1±19.3	133.3±9.0	221.9±44.6	212.4±31.0
Mn, μg/g	157.7±12.1	88.0±11.1	142.5±20.1	47.4±4.8	786±125.7	735±100.1	304.0±44.4	328.0±31.0
Zn, μg/g	63.4±4.5	81.7±4.7	58.3±5.0	77.4±6.9	119.6±7.9	148.5±12.6	97.1±8.4	126.8±12.1
Cu, μg/g	6.03±0.43	6.50±0.34	4.78±0.40	5.89±0.52	5.87±0.32	7.98±0.60	6.49±0.78	8.90±1.16
