# Supplementary material for: Mineral Nutrition of Naturally Growing Scots Pine and Norway Spruce under Limited Water Supply
Source: Plants (Basel). 2022 Oct 9;11(19):2652. doi: 10.3390/plants11192652 (PMC9573269; doi:10.3390/plants11192652)
Supplement: Supplementary file 1 [file plants-11-02652-s001.zip › Table S3.pdf]

Table S3. Contents of lipid peroxidation products and photosynthetic pigments in pine and spruce needles.

Parameter	Pine Needles				Spruce Needles			
	Normal (Site II)		Arid (Site III)		Normal (Site I)		Arid (Site II)	
	N3	N24	A3	A24	N3	N24	A3	A24
MDA, nmol/g	52.8±1.6	59.2±2.6	47.5±2.3	60.4±2.3	31.6±6.2	49.9±3.7	36.2±3.7	28.7±1.2
4-HNE, nmol/g	1145±64.2	1074±72.4	1119±116.1	1324±90.7	918±126.7	830±91.1	911±269.8	535±42.8
Chl a, mg/g	2.39±0.23	1.88±0.09	2.48±0.16	2.21±0.11	2.69±0.14	2.93±0.18	2.38±0.13	2.17±0.07
Chl b, mg/g	0.94±0.09	0.73±0.04	1.01±0.06	0.89±0.06	1.15±0.06	1.22±0.08	0.99±0.06	0.90±0.03
Car, mg/g	0.42±0.03	0.35±0.01	0.42±0.03	0.40±0.01	0.43±0.02	0.47±0.03	0.37±0.02	0.36±0.01
Chl a/Chl b	2.54±0.02	2.56±0.02	2.46±0.01	2.51±0.04	2.35±0.02	2.41±0.03	2.41±0.02	2.41±0.02
Car/Chls	0.13±0.00	0.14±0.00	0.12±0.00	0.13±0.00	0.11±0.00	0.11±0.00	0.11±0.00	0.12±0.00
